# Liposome Lipid-Based Formulation Has the Least Influence on rAAV Transduction Compared to Other Transfection Agents

**DOI:** 10.1016/j.omtm.2018.04.004

**Published:** 2018-04-12

**Authors:** Pengpeng Guo, Chenghui Yu, Qingxin Wang, Ruirong Zhang, Xianze Meng, Yinglu Feng

**Affiliations:** 1 Institute of Integrative Medicine, Qingdao University Medical College, Qingdao, Shandong 266021, China; 2 Department of Genetics, School of Life Sciences, Fudan University, Shanghai 200438, China; 3 Department of Traditional Chinese Medicine, PLA 401 Hospital, Qingdao City, Shandong Province FL 266071, China

**Keywords:** liposome lipid-based formulation, rAAV, transduction, transfection agents, trichosanthin

## Abstract

Recombinant adeno-associated virus (rAAV) vectors are considered ideal vehicles for human gene therapy. Meanwhile, non-viral strategies, such as transfection agents (TAs), have also shown promise to deliver genetic materials, such as siRNA. Transduction with the rAAV vector is performed concurrently with transfection with plasmid DNA or RNA. In the present study, we report that various TAs inhibited rAAV-mediated transgene expression at diverse levels. Overall, cationic polymers and dendrimers dramatically blocked rAAV transduction, while lipid-based liposomes displayed the least effect. The inhibitory effect was dependent on the dose of TAs and the timing of infection, suggesting that the early stages of viral infection were involved. In addition, the present results indicate that the transgene expression of rAAV vectors was significantly increased by liposome-mediated transfection with adenoviral helper genes. At the same time, this was dramatically inhibited by liposome-mediated transfection with the trichosanthin gene encoding a type I ribosome-inactivating protein isolated from traditional Chinese medicine. Furthermore, liposomes also have little effect on rAAV-mediated transgene expression *in vivo*. Taken together, these findings suggest liposome as the best choice of TAs, which should be used in combination with rAAV-mediated gene therapy.

## Introduction

The ability to introduce DNA into cultured cells has provided a powerful means to study the function and control of mammalian genes. To date, various techniques have been used to deliver DNA into mammalian cells, including viral vectors, nanoparticles, and transfection agents (TAs). Among all of these techniques, TAs are the most frequently used methods in laboratory for both transient and stable transfection of a variety of cell types. There are various classes of TAs, including, but not limited to, cationic polymers, cationic lipids, dendrimers, and calcium phosphate-base agents.

Calcium-phosphate-mediated transfection involves mixing DNA directly with CaCl_2_ and a phosphate buffer to form a fine precipitate, which is dispersed over cultured cells.^1^ It is a very inexpensive technique yet time consuming to perform. The preparation of the DNA mixed solution usually takes 20–40 min. Another limitation is the resulting transfection efficiency. The number of cells that express the desired gene is usually quite limited, which is <20% in most cases.

Cationic polymers, such as polyethylenimines (PEIs), have recently been intensively used for gene transfer. Both long, branched PEIs (>800 kDa)^2^ and low-molecular-weight linear PEIs (< 25 kDa)^3^ have been used to transfect mammalian cells. The latter is of particular interest due to its stability in physiological fluids such as serum and its relatively high transfection efficiency both *in vitro* and *in vivo*. The PEI-DNA complexes escape from the endosomes and de-condense in the cytoplasm as early as 4 hr after introduction into cells.^4^

Polyamidoamine dendrimers represent another family of cationic polymers, which have been routinely utilized to deliver DNA into cell cultures since the late 1990s.^5^ *In vivo* studies have also performed the delivery of DNA to the carotid artery,^6^ heart,^7^ and lungs.^8^ Dendrimers possess a defined spherical architecture, with branches radiating from a central core and terminating at charged amino groups. These assemble DNAs into compact structures, optimizing the entry of DNA into a cell. Dendrimer-DNA complexes possess a net positive charge, which allows these to bind to negatively charged receptors (e.g., sialylated glycoproteins) on the surface of eukaryotic cells and subsequently enter cells.

Another popular chemical transfection methodology includes the use of either cationic liposome-based (e.g., Lipofectamine 2000, Invitrogen) or non-liposome-based (e.g., Effectene, QIAGEN) lipids. The basic structure of cationic lipids consists of a hydrocarbon chain and a positively charged head group, which ensures the interaction between the lipid and phosphate backbone of the nucleic acid. The transfection complex has been considered to interact with the negatively charged cell membrane and enter cells through endocytosis.^9^ However, the exact mechanism of DNA release from endosomes and those of the subsequent translocation to the nucleus remain unclear. Generally, cationic lipid-agent-mediated transfection yields high efficiencies in a wide variety of eukaryotic cells, especially those resistant to transfection through other methods.

All of the aforementioned TAs have been used to study various steps in adeno-associated virus (AAV) life cycles. For instance, Liu et al. transfected HEK293 cells with DsRed-Rab5, -Rab7, or -Rab11 expression plasmids using the calcium phosphate precipitation method and revealed that that late endosomes might not be involved in the transduction pathway of AAV8.^10^ Meanwhile, Nonnenmacher and Weber utilized cationic polymers (GeneJammer, Agilent Technologies) to deliver various genes into HeLa cells and concluded that AAV2 uses the pleiomorphic clathrin-independent carriers/GPI-anchored-protein-enriched endosomal compartment (CLIC/GEEC) pathway as its major endocytic infection route.^11^ On the other hand, Srivastava et al. extensively studied the roles of a cellular serine/threonine protein phosphatase, protein phosphatase 5 (PP5), in rAAV2-mediated gene transfer. Most of their experiments used liposome-based lipids.^12^, ^13^, ^14^, ^15^

In some instances, the transduction with AAV vectors was performed concurrently with transfection with plasmid DNA or RNA. For example, the therapeutic applications of RNAi can be achieved by both transfection agents^16^ and AAV vectors.^17^ Another example is the use of AAV vectors to deliver donor templates, together with transfection agent-mediated DNA or RNA, in order to deliver Cas9. However, no systematic studies have compared the effectiveness of these chemical transfection systems on rAAV-mediated transgene expression. The purpose of the present study was to determine the effects of different TAs, either alone or in combination with plasmids at various time points, on rAAV2 transduction efficiency. An optimized strategy that minimizes the inhibition from TAs and truly reflects the contribution of transgene products is recommended.

## Results

### Effect of Various TAs on rAAV2 Vector-Mediated Transgene Expression in the Absence of Plasmid DNA

**Figure 1:**
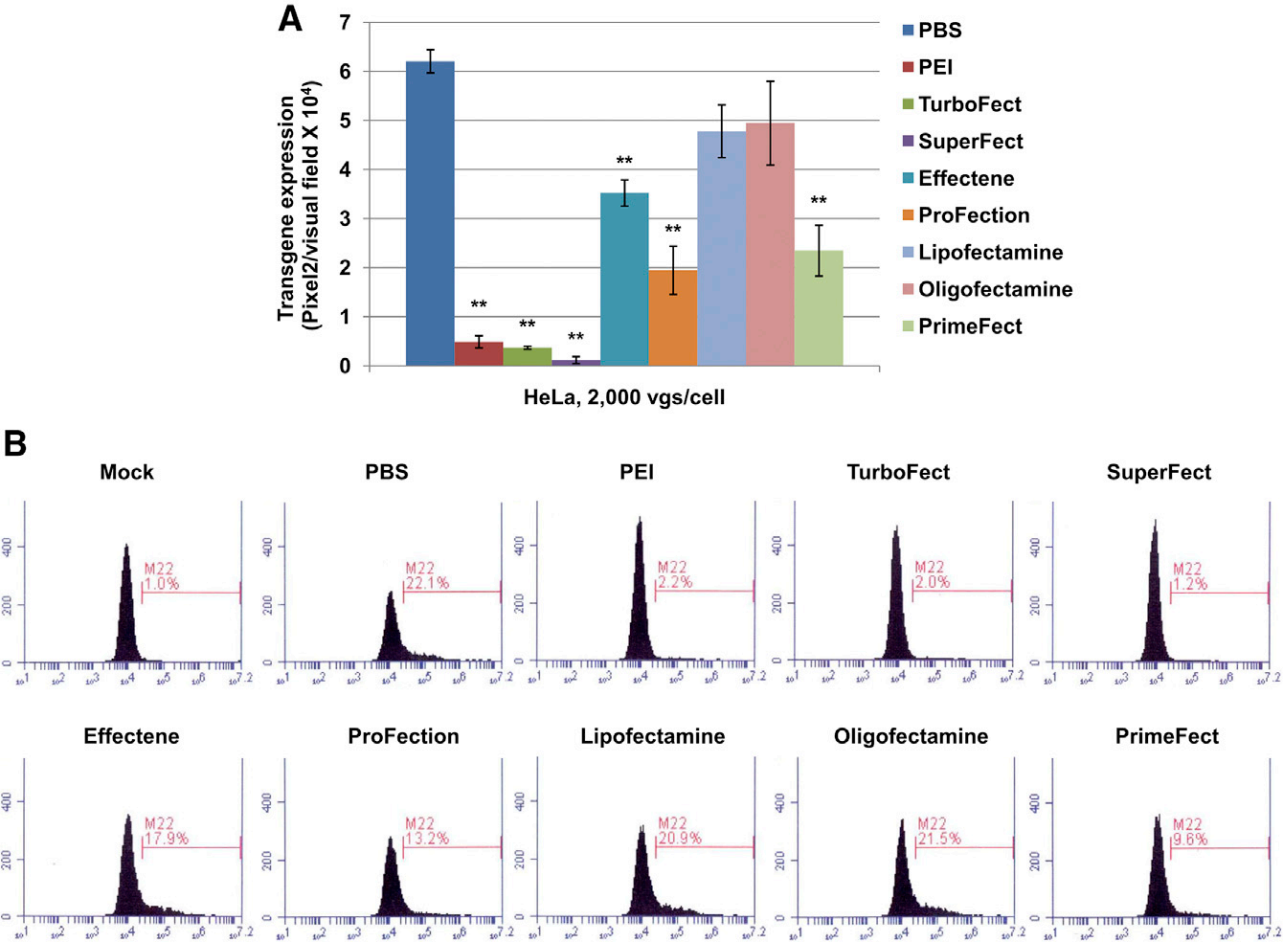
The rAAV2-Mediated Transgene Expression in the Presence of Various Transfection Reagents (A) HeLa cells were treated with different transfection reagents for 6 hr without plasmids, according to the company’s manual. Then, cells were washed with PBS twice and transduced with scAAV2-CBAp-*egfp* at 2,000 vgs per cell. Transgene expression was detected by fluorescence microscopy at 72 hr post-transduction. (B) The transgene expression was measured by flow cytometry at 72 hr post-transduction. The results are presented as mean ± SD. **p < 0.01 versus PBS.

In the first set of experiments, a number of TAs were used to treat HeLa cells without plasmids, followed by infection with self-complementary (sc) AAV2 vectors carrying EGFP driven by a chicken β-actin/cytomegalovirus (CMV) hybrid promoter (CBAp) at an MOI of 2,000 viral genomes (vgs) per cell. As shown in Table 1, PEI and TurboFect are cationic polymers, SuperFect is a specifically designed heat-activated dendrimer, Effectene is a non-liposomal lipid formulation, ProFection is based on calcium phosphate, and Lipofectamine and Oligofectamine are lipid-based liposomes. Unfortunately, the components of PrimeFect from Lonza were not released. It was evident that all TAs inhibited rAAV2-mediated transgene expression at some level (Figure 1A), in which PEI, TurboFect, and SuperFect dramatically blocked rAAV2 transduction. On the other hand, lipid-based liposomes (Lipofectamine and Oligofectamine) slightly reduced rAAV2 transduction. The blocking effects of Effectene, ProFection and PrimeFect were in the middle among all tested TAs. Figure 1B shows the results of the flow cytometry analysis, which further supports these conclusions. The percentage of EGFP-positive cells in the PBS treatment group was approximately 22%. Although PEI, TurboFect, and SuperFect treatment reduced the percentage to a level close to that of mock infection, both Lipofectamine and Oligofectamine treatment had no significant effect (approximately 21% and 22%, respectively).

**Table 1:** Mechanisms of Different Transfection Reagents

Name	Composition	Mechanism
PEI	cationic polymer	condenses DNA into positively charged particles; binds to anionic cell surface residues; enters the cell via endocytosis
TurboFect	cationic polymer	
SuperFect	dendrimer	condenses DNA into positively charged particles; binds to negatively charged cellular surface receptors; inhibits lysosomal pH after fusion with the endosome
Effectene	non-liposomal lipid formulation	unknown
ProFection	calcium phosphate	mixes DNA directly with CaCl_2_ and a phosphate buffer; forms a fine precipitate; disperses over the cultured cells
Lipofectamine	lipid formulation	forms liposomes and entraps DNAs; fuses with the plasma membrane
PrimeFect	unknown	unknown

### The Dose- and Time-Dependent Influence of SuperFect on rAAV2 Vector-Mediated Transgene Expression

**Figure 2:**
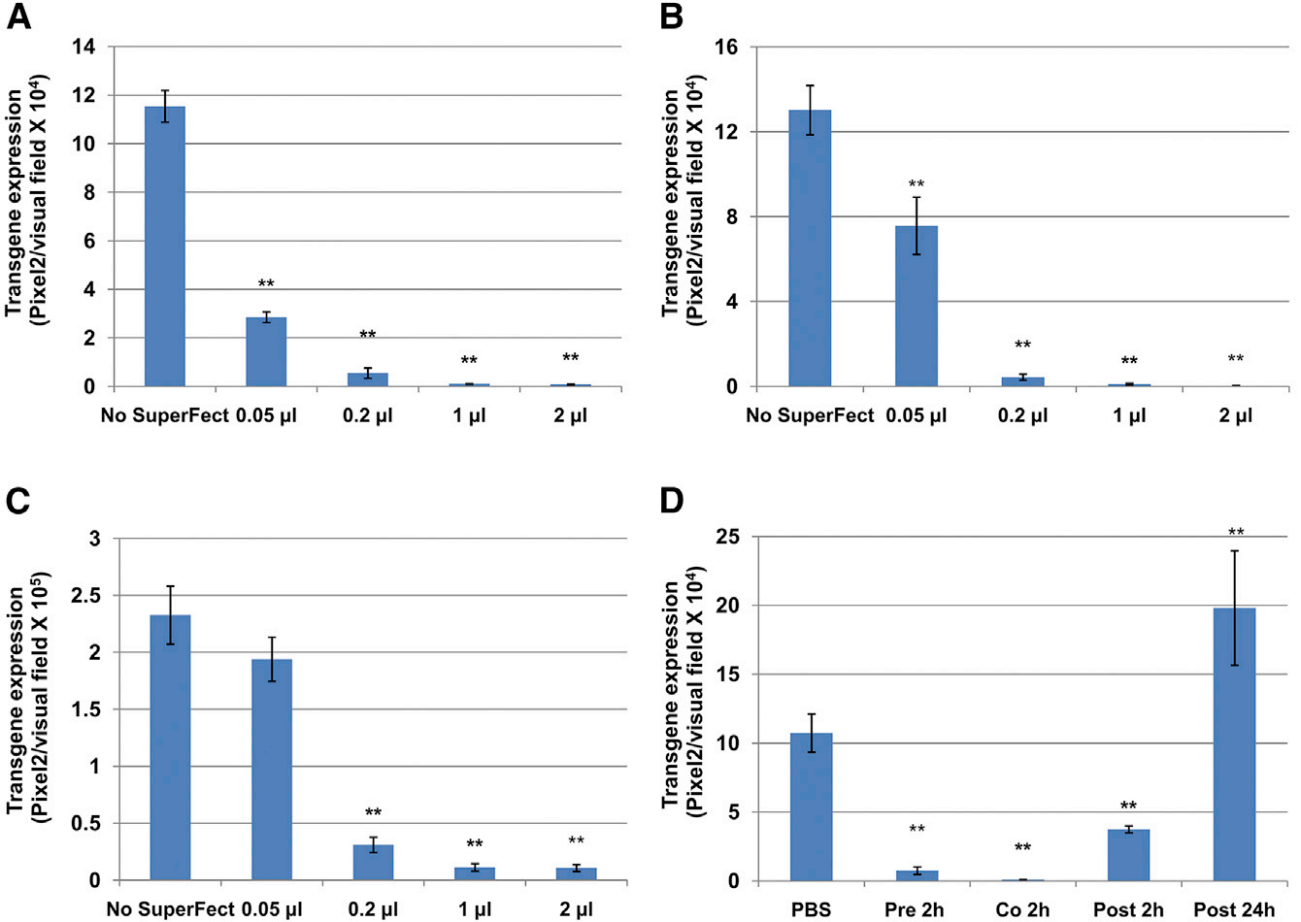
The Effect of SuperFect on rAAV2-Mediated Transgene Expression Cells were treated with SuperFect at various doses for 6 hr without plasmids and washed with PBS twice. (A) HeLa cells were transduced with scAAV2-CBAp-*egfp* at 2,000 vgs per cell. (B) HeLa cells were transduced with ssAAV2-CMVp-*hrgfp* at 2,000 vgs per cell. (C) HEK293 cells were transduced with scAAV2-CBAp-*egfp* at 2,000 vgs per cell. (D) HeLa cells were treated with SuperFect at various time points without plasmids, together with the transduction with scAAV2-CBAp-*egfp* at 5,000 vgs per cell for 2 hr. Transgene expression was detected by fluorescence microscopy at 72 hr post-transduction. The results are presented as mean ± SD. **p < 0.01 versus PBS.

Since SuperFect was the most dramatically observed to inhibit scAAV2 transduction among all tested TAs, the investigators attempted to further confirm this observation in other conditions. HeLa cells were seeded in a 96-well plate and treated with SuperFect at various doses for 6 hr. It is worth noticing that 2 μL SuperFect for each of the 96 wells was recommended by the manufacturer for normal transfection assays.^18^ As shown in Figure 2A, a SuperFect treatment of as low as 0.05 μL significantly blocked scAAV2-mediated transgene expression. The same effect was also observed, regardless of the target cell types (HeLa versus HEK293) or vg types (single stranded versus self-complementary) (Figures 2B and 2C). Interestingly, although the pre-treatment and co-treatment of cells with SuperFect dramatically inhibited rAAV2 transduction, exposure to TA at 2 hr post-viral infection partially rescues its negative effect. Moreover, treatment with SuperFect at 24 hr post-viral infection significantly enhanced viral transduction, which suggests that the TA’s inhibition effect is most likely due to the interference of early stages during viral infection (Figure 2D).

### Effect of Various TAs on rAAV2 Vector-Mediated Transgene Expression in the Presence of Plasmid DNA

**Figure 3:**
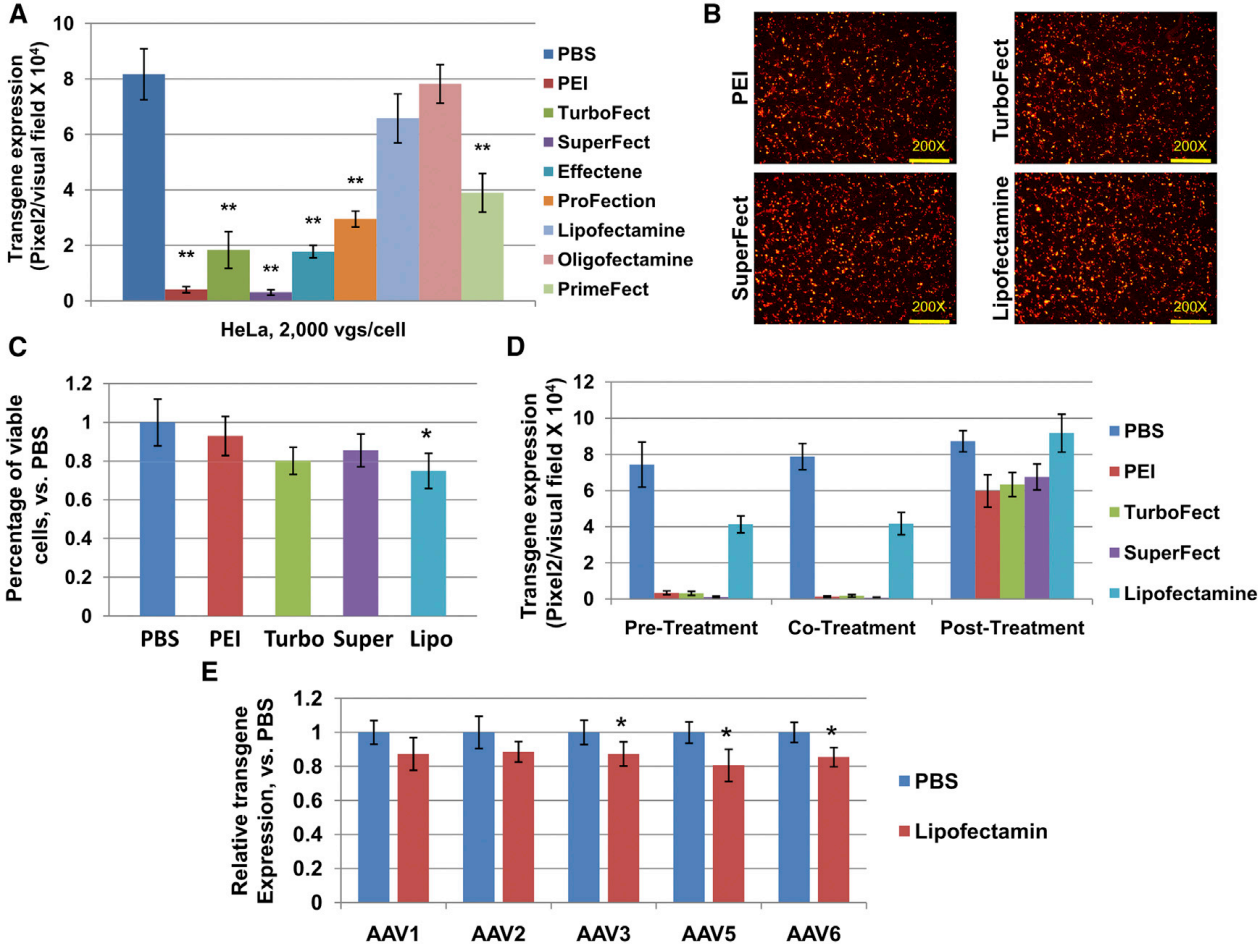
The rAAV2-Mediated Transgene Expression following Plasmid Transfection (A–D) HeLa cells were transfected with plasmid pAAV-*fluc*-2A-*mapple* using different TAs for 6 hr, (A) according to the company’s manual or (B and E) with slightly changed protocols. Then, cells were washed with PBS twice and transduced with scAAV2-CBAp-*egfp* at 2,000 vgs per cell. (A) Viral transduction was determined by fluorescence microscopy at 72 hr post-transduction. (B) The transfection efficiency of pAAV-*fluc*-2A-*mapple* was determined by fluorescence microscopy at 72 hr post-transduction; original magnification, ×200. (C) The percentage of viable cells was determined at 72 hr post-transduction by CCK8. (D) Viral transduction was determined by fluorescence microscopy at 72 hr post-transduction. (E) Cells were transfected with plasmid by Lipofectamine, followed by transduction with various serotype vectors carrying CBAp-*egfp* at 2,000 vgs per cell. Viral transduction was determined by fluorescence microscopy at 72 hr post-transduction. The results are presented as mean ± SD. *p < 0.05 versus PBS; **p < 0.01 versus PBS.

Considering that TAs are commonly utilized together with plasmids, systemic comparison was subsequently carried out to determine the influence of variant TAs with plasmid on rAAV2-mediated transgene expression. Plasmid pAAV-*fluc*-2A-*mapple* carries both Firefly luciferase and mApple genes, which are separated by a 2A sequence^19^ and driven by a CBAp. HeLa cells were subjected to transfection assays, according to the manufacturer’s instructions, and infected with scAAV2-CBAp-*egfp* vectors. The results presented in Figure 3A demonstrated a similar trend of effects, in which cationic polymers and dendrimers dramatically blocked rAAV2 transduction and, at the same time, lipid-based liposomes only slightly affected the transgene expression. Furthermore, identical results were also observed when plasmid pAAV-CBAp-*egfp* was transfected into cells and viral vector ssAAV2-*fluc*-2A-*mapple* was used to infect cells (data not shown). However, due to the inherent efficiency of transfection, it is worth noticing that the expression of genes from the transfection assays, according to the manufacturer’s instruction, varies among all tested TAs. To this end, in the following experiments, the investigators mainly focused on the four most efficient TAs (PEI, TurboFect, SuperFect, and Lipofectamine 3000), and slightly adjusted the amount of plasmids in each transfection assay in order to result in a similar transgene expression from the plasmids (Figure 3B). The results of the cell viability assays presented in Figure 3C indicate that all transfection agents slightly reduced cell viability (p > 0.01). Next, HeLa cells were transduced with scAAV2-CB-*egfp* vectors and transfected with adjusted doses of pAAV-*fluc*-2A-*mapple* at various time points (Figure 3D). When transfection assays were performed at pre- or co-infection, all TAs significantly inhibited viral transduction, in which Lipofectamine possessed the least effect. Interestingly, when transfection assays were performed at 2 hr post-infection, the negative effects were largely rescued. Although PEI, TurboFect, and SuperFect slightly inhibited viral transgene expression, it did not reach a statistically significant result. In addition, several viral serotypes, including AAV1, AAV2, AAV3, AAV5, and AAV6, were tested. The results presented in Figure 3E indicates that Lipofectamine-mediated transfection with plasmid has a moderate effect on all test vectors.

### Lipofectamine-Mediated Plasmid DNA Transfection Influences the Extent of rAAV Vector-Mediated Transgene Expression

**Figure 4:**
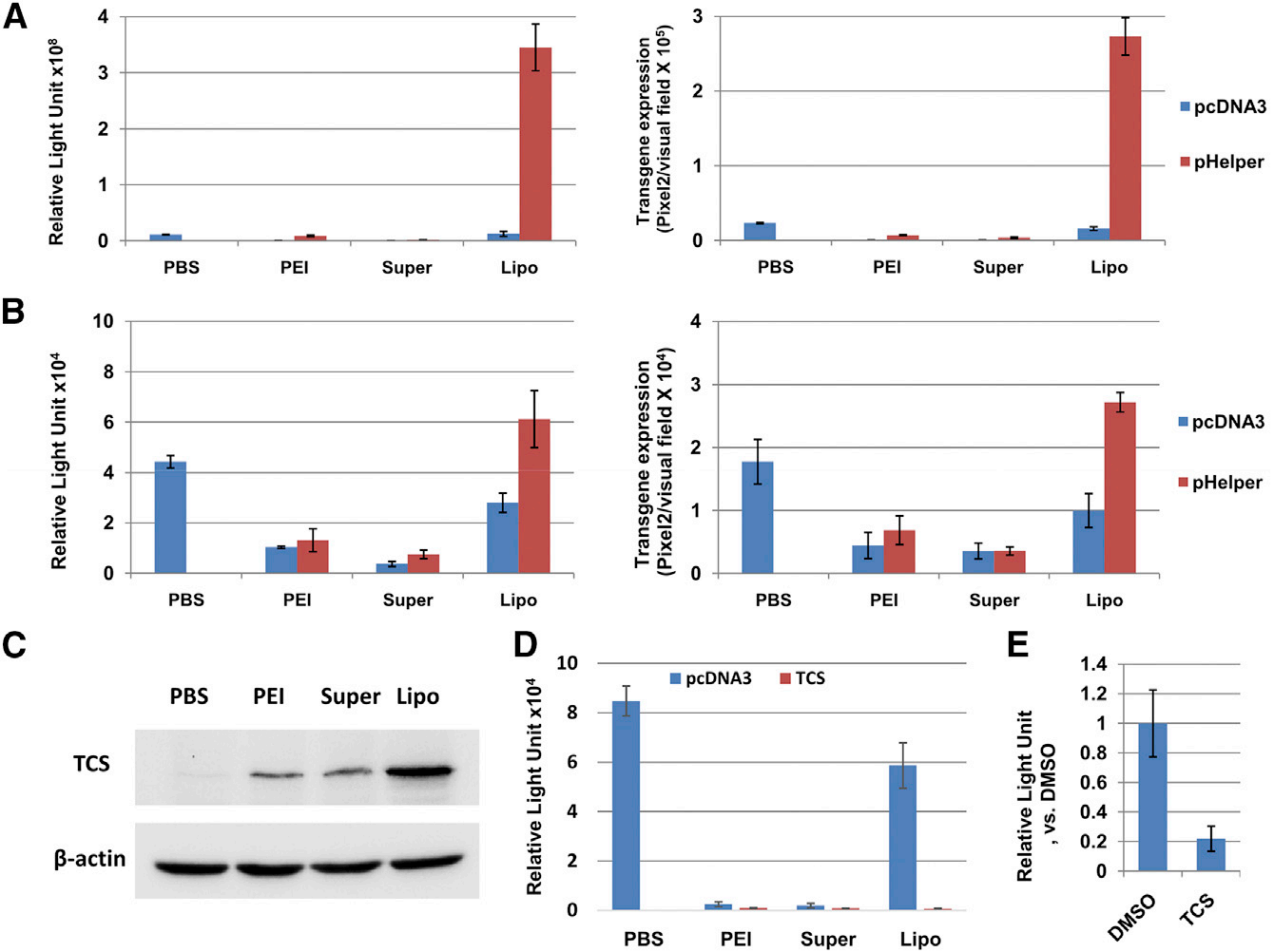
The Roles of the Adenoviral Genes and Cellular Ribosomal Activity in rAAV-Mediated Transgene Expression (A) HeLa cells were transfected with pHelper plasmids, followed by transduction with ssAAV2-*fluc*-2A-*mapple* vectors at 2,000 vgs per cell. The FLuc expression (left) and mApple expression (right) were determined at 72 hr post-viral transduction. (B) HeLa cells were transduced with scAAV2-*fluc*-2A-*mapple* vectors at 2,000 vgs per cell, followed by transfection with pHelper plasmids. FLuc expression (left) and mApple expression (right) were determined at 72 hr post-viral transduction. (C and D) HeLa cells were transfected with trichosanthin-expressing plasmid, followed by transduction with ssAAV2-*fluc*-2A-*mapple* vectors at 2,000 vgs per cell. (C) The expression of trichosanthin was determined by western blot assay at 48 hr post-transfectoin. (D) The FLuc expression was determined at 72 hr post-viral transduction. (E) HeLa cells were treated with trichosanthin protein at 25 μM, followed by transduction with ssAAV2-*fluc*-2A-*mapple* vectors at 2,000 vgs per cell. The FLuc expression was determined at 72 hr post-viral transduction. The results are presented as mean ± SD.

In order to apply these present observations in AAV studies, these experiments were conducted using plasmid pHelper for transfection and viral vector ssAAV2-*fluc*-2A-*mapple* for infection. pHelper contains three genes from adenovirus that have been shown to influence various steps during ssAAV2 infection. The ssAAV2-*fluc*-2A-*mapple* vector was chosen because it carries both Firefly luciferase and mApple genes, and because its length is close to that of wild-type AAV2, which makes the present conclusion more convincing. First, HEK293 cells were transduced with viral vectors, together with the transfection with plasmids using various TAs. It was evident that, compared to control plasmid pcDNA3, only Lipofectamine-mediated transfection with plasmid pHelper resulted in a >10-fold increase in ssAAV2-mediated transgene expression (Figure 4A). Furthermore, the HeLa cell line was also tested to further support these present conclusions. Compared to co-transfection with control plasmid pcDNA3, only Lipofectamine-mediated co-transfection with plasmid pHelper significantly enhanced ssAAV2-mediated transgene expression (Figure 4B).

Trichosanthin (TCS) is found in the herb *Trichosanthes kirilowii*, which functions to clear body heat and reduce body fire in the traditional Chinese medicine theory.^20^ Its enzymatic activity is recognized as a type I ribosome-inactivating protein.^21^ In order to evaluate whether the rAAV-mediated transgene expression is dependent on ribosomal activity, a TCS-expressing plasmid was transfected into HEK293 cells using either PEI, SuperFect, or Lipofectamine. The expression of TCS protein was confirmed by western blot assays (Figure 4C). Following plasmid transfection, the cells were infected with ssAAV2-*fluc*-2A-*mapple* vectors. It was evident that the PEI- and SuperFect-mediated transfection of TCS led to the 3-fold (p < 0.05) and 2-fold (p > 0.05) reductions of viral transgene expression, respectively. On the other hand, Lipofectamine-mediated TCS expression resulted in the dramatic reduction (>50-fold) of viral transgene expression, suggesting that the rAAV-mediated transgene expression is highly dependent on ribosomal activity. In order to further corroborate these results, cells were treated with TCS protein, followed by rAAV transduction. Similar results were observed, showing that TCS significantly reduced rAAV-mediated transgene expression (Figure 4E). Taken together, it was concluded that liposome-based transfection truly reflects the contribution of transgene products and should be recommended for AAV studies.

### Cationic Liposome Lipid-Based Formulation Has a Minimal Effect on rAAV-Mediated Transgene Expression *In Vivo*

**Figure 5:**
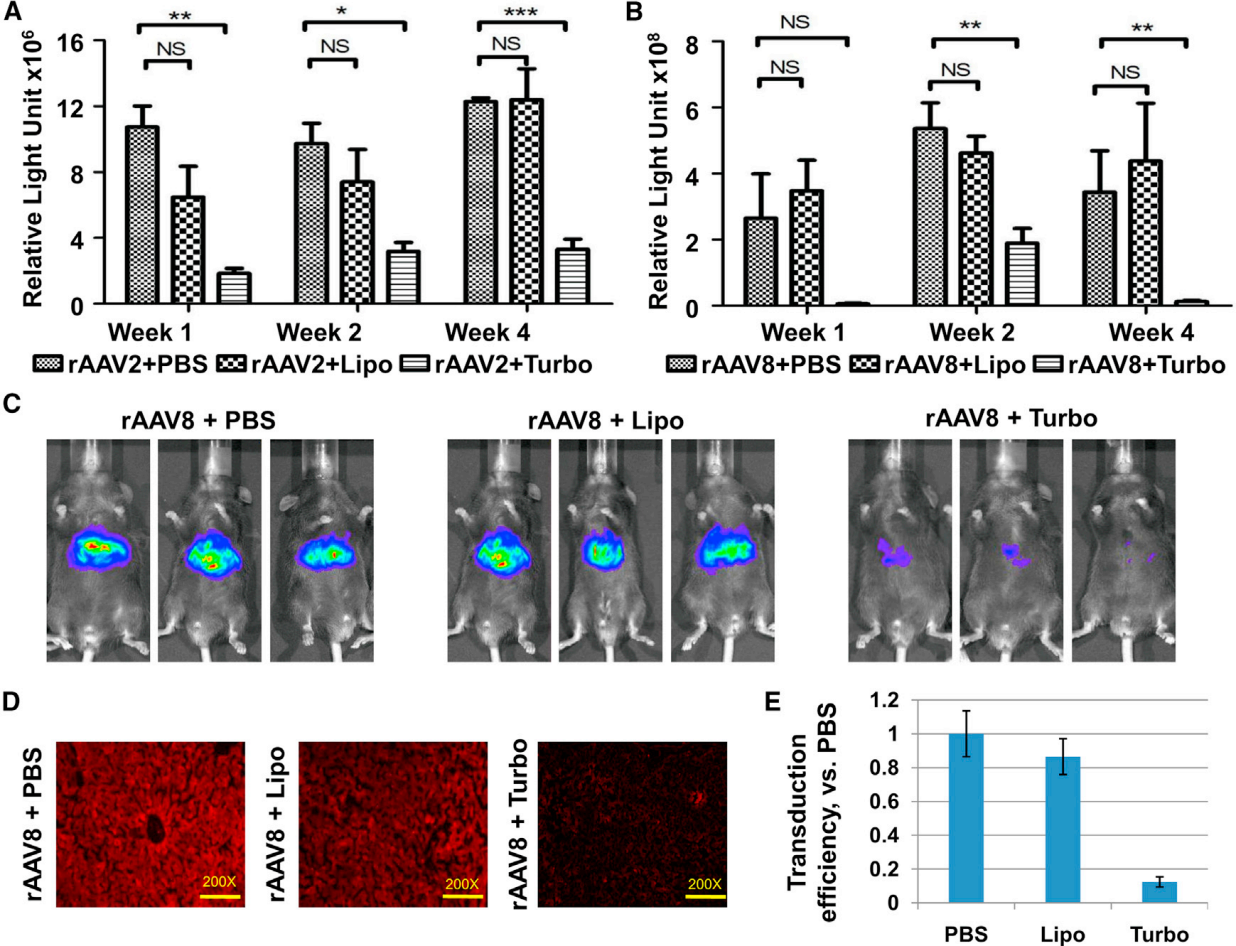
The Transduction Efficiency of rAAV2 and rAAV8 Vectors in Mouse Liver in the Presence of *In Vivo* TAs C57BL/6 mice (n = 3) were injected with 10^12^ viral genomes per animal of either ssAAV2-*fluc*-2A-*mapple* or ssAAV8-*fluc*-2A-*mapple* vectors, together with a liposome lipid-based liver *in vivo* TA (Altogen Biosystems) or a TurboFect-based *in vivo* TA (Thermo Scientific). Live images were obtained at various time points at post-vector administration to determine the luciferase activity. (A and B) Quantitation of the signal intensity in each individual mouse. (A) rAAV2 vectors. (B) rAAV8 vectors. (C) The visual output represents the number of photons emitted per second per square centimeter as a false color image, in which the maximum is red and the minimum is blue. (D) At the endpoint of the experiment, the livers were harvested, sectioned, and analyzed using a Leica DM-5500 microscope. Representative images of the liver sections were presented; original magnification, ×200. (E) The quantitative data of the fluorescent images from liver sections. The results are presented as mean ± SD. *p < 0.05 versus PBS; **p < 0.01 versus PBS; ***p < 0.01.

Given that both rAAV vectors and liposome-based strategies resulted in promising results *in vivo*, and that the transduction with AAV vectors is expected to be performed concurrently with the transfection with plasmid DNA or RNA, the investigators attempted to extend their observation to animal studies. To this end, C57BL/6 mice were tail-vein injected with ssAAV-*fluc*-2A-*mapple* vectors, together with plasmid pcDNA3, using either a TurboFect *in vivo* transfection reagent (Thermo Scientific) or a liposome lipid-based liver transfection reagent (Altogen Biosystems, Las Vegas, NV, USA), according to the manufacturer’s instructions. Then, the transgene expression of Firefly luciferase was determined at 1 week, 2 weeks, and 4 weeks post-viral injection. It was evident that the rAAV8 vectors (Figure 5A) resulted in an approximately 20-fold higher transduction efficiency, compared with the rAAV2 vectors (Figure 5B), which is consistent with previous reports. Most importantly, the cationic polymer dramatically inhibited viral-mediated transgene expression, regardless of the rAAV serotypes. On the other hand, the liposome lipid-based formulation has little effect on the viral-mediated transgene. Representative images of *fluc* gene expression in the liver, presented in Figure 5C, suggest that liposome lipid-based formulation has little effect on the viral bio-distribution. Furthermore, at the endpoint of the experiment, mice were sacrificed, and liver tissues were harvested to determine the expression of mApple. These results were consistent with what was observed using a bioluminescence imaging system, indicating that the cationic polymer, and not the liposome, significantly inhibited viral-mediated transgene expression in the liver (Figure 5D).

## Discussion

Recombination AAV vector is considered an ideal vehicle for human gene therapy, which is mainly attributed to its safety profile and high efficiency. However, there are a number of factors that can affect the transduction capacity of AAV vectors. In order to investigate these cellular contributors, various transfection agents have been used together with rAAV transduction. However, the potential impact on rAAV-mediated transgene expression remains largely unknown. Moreover, in some cases, rAAV vectors need to be delivered into mammalian cells together with the DNA. For instance, in a recent study of targeted gene knockin, DNA that expressed a single guide RNA was required to induce a double-strand break that stimulates the DNA repair pathway, while the rAAV vector was used to mediate the delivery of a homology-directed repair donor template.^22^ Therefore, the aim of the present study was to determine whether different TAs would affect rAAV transduction efficiency. No comprehensive studies have been reported pertaining to the inhibiting action on rAAV induced by TAs.

The present data indicated that different TAs inhibit rAAV2-mediated transgene expression at different levels. Overall, PEI, TurboFect, and SuperFect dramatically block rAAV2 transduction, whereas lipid-based liposomes display the least effect. These results are coincident, both in the presence of and in the absence of plasmids. In addition, both *in vitro* and *in vivo* studies have suggested that the use of liposome lipid-based formulation should be recommended in rAAV research. Furthermore, the present data also illustrated that the inhibition effect of TA was most likely due to the interference of early stages during viral infection. The main conclusion from the present results is that liposome-based gene delivery strategy could be used together with AAV-mediated gene therapy. In order to draw a solid conclusion, the experiments were designed in two ways. On one hand, the expression of adenoviral genes was expected to enhance AAV transduction. On the other hand, the expression of TCS was expected to reduce AAV transduction.

Effective rAAV transduction involves the following steps: virus binding on the target cell surface, receptor-mediated endocytosis into endosomes and lysosomes, cytoplasmic traffic, nuclear import, capsid protein degradation, and second-strand synthesis before transgenic expression. In the endocytic system, rAAV vectors were trafficked to lysosomes through early endosomes, late endosomes, and recycling endosomes.^23^ Under endosomal acidification, the vectors expose the N-termini of viral capsid proteins to release a conserved phospholipase A2 (PLA2) motif, which is critical for the viral escape from endosomes to the cytoplasm. This conformational change occurs in an acidic environment.^24^ In an effort to explain the potential mechanism of the inhibitory effect of TAs, the investigators noticed that SuperFect is a specifically designed activated dendrimer. It assembles DNA into compact structures and possesses a net positive charge, which allows them to bind to negatively charged receptors on the surface of eukaryotic cells. Once inside, it buffers the lysosome after it has fused with the endosome, leading to the pH inhibition of lysosomal nucleases and the stability of SuperFect-DNA complexes.^25^ Unfortunately, the reasons why other TAs inhibited rAAV vector transduction remain unclear. Nevertheless, the present findings may explain the negligible amounts of homology-directed repair (HDR) when the donor templates were delivered by rAAV vectors, while the nuclease expression cassettes were through other means in previous researches.^22^, ^26^, ^27^, ^28^

In summary, the present study provides a strategy to optimize the experimental procedure of using an established approach based on rAAV. The aforementioned measures are for maximizing the efficiency of virus infection, and further efforts to test and determine the optimal time point of infection may contribute to its improvement.

## Materials and Methods

### Cell Lines and Cultures

Human cervical cancer HeLa and HEK293 cell lines were purchased from the American Type Culture Collection (Manassas, VA, USA) and maintained in complete DMEM (Mediatech, Manassas, VA, USA), supplemented with 10% heat-inactivated fetal bovine serum (FBS; Sigma-Aldrich, St. Louis, MO, USA), and 1% penicillin and streptomycin (P/S; Lonza, Walkersville, MD, USA). Cells were grown as adherent cultures in a humidified atmosphere at 37°C in 5% CO_2_, sub-cultured after treatment with a trypsin-versene mixture (Lonza, Walkersville, MD, USA) for 2–5 min at room temperature, and washed and re-suspended in complete medium.

### Plasmid Transfection

The pHelper plasmid and TCS plasmid were prepared as previously described.^29^ Cells were seeded in 96-well plates at a concentration of 1 × 10^4^ cells per well in complete medium before transfection. After 24 hr of incubation in complete DMEM, the cells were subjected to transfection assays, according to the manufacturer’s instructions.

Briefly, the following procedures were performed.

#### PEI

Linear PEIs (Polysciences, catalog #23966) were diluted in PBS at a concentration of 1 mg/mL (pH 4.5). The plasmids were pre-mixed with DMEM without FBS and antibiotics, mixed with PEI, and incubated at room temperature for 5 min. Then, the DNA-PEI complexes were added to cells growing in complete DMEM.

#### TurboFect

Plasmids were pre-mixed with DMEM without FBS and antibiotics, mixed with TurboFect (Thermo Scientific, catalog #R0531), and incubated at room temperature for 15 min. Then, the DNA-TurboFect complexes were added to cells growing in complete DMEM.

#### SuperFect

Plasmids were pre-mixed with DMEM without FBS and antibiotics, mixed with SuperFect (QIAGEN, catalog #301305), and incubated at room temperature for 5 min. Then, the DNA-SuperFect complexes were added to cells growing in complete DMEM.

#### Effectene

Plasmids were pre-mixed with DNA condensation buffer and enhancer, mixed with Effectene transfection reagent (QIAGEN, catalog #301425), and incubated at room temperature for 5 min. Then, the DNA-Effectene complexes were added to cells growing in complete DMEM.

#### ProFection

The ProFection mammalian transfection system was purchased from Promega. The plasmids were pre-mixed with CaCl_2_, slowly mixed with 2× Hank’s balanced salt solution (HBSS), and incubated at room temperature for 30 min. Then, DNA-CaCl_2_ precipitates were added to cells growing in complete DMEM.

#### Lipofectamine and Oligofectamine

Plasmid DNA and Lipofectamine 2000 (Invitrogen, catalog #11668) and Oligofectamine (Invitrogen, catalog #12252) were diluted in two tubes of DMEM without FBS and antibiotics. After 5 min of incubation at room temperature, DNA and Lipofectamine/Oligofectamine were combined and incubated for an additional 20 min at room temperature. Then, the DNA-Lipofectamine/Oligofectamine complexes were added to cells growing in complete DMEM.

#### PrimeFect

Plasmid DNA and PrimeFect I (Lonza, catalog #PA-3267) were diluted in two tubes of DMEM without FBS and antibiotics. After 5 min of incubation at room temperature, DNA and PrimeFect were combined. Then, the DNA-PrimeFect complexes were immediately added to cells growing in complete DMEM.

Each experiment was carried out in triplicate and repeated three times. Following transfection, cells were incubated at 37°C in humidified air (5% CO_2_) for 6 hr. Then, the transfection medium was removed, and the cells were incubated for an additional 48 hr in complete DMEM.

### Recombinant AAV2 Vector Production

The rAAV vectors were produced with 10E−13 vgs/mL by Omnivecter, Shanghai, China. Briefly, HEK293 cells were transfected with three plasmids using PEI (linear, molecular weight [MW], 25,000; Polysciences), as previously described. At 72 hr post-transfection, cells were harvested, and the vectors were purified by iodixanol (Sigma) gradient centrifugation and ion exchange column chromatography (5 mL HiTrap SP HP, GE Healthcare). Then, the virus was concentrated, and the buffer was exchanged in three cycles to lactated Ringer’s solution using centrifugal spin concentrators (Apollo, 150 kDa cutoff, 20 mL capacity, CLP). The entire packaging processes were performed as previously described.^30^ The physical particle titers highly purified of rAAV2 vector stocks were determined by quantitative DNA slot-blot and Southern blot analyses, as previously described.^31^

### The rAAV2 Vector Transduction Assays *In Vitro*

Cells were seeded in 96-well plates at a concentration of 1 × 10^4^ cells per well in complete medium and were transduced with AAV vectors at 5,000 vgs per cell in DMEM without FBS and antibiotics for 2 hr. After transduction, the cells were washed by PBS twice and incubated for an additional 72 hr in complete DMEM. Then, the expression of the reporter genes was analyzed by direct fluorescence imaging, flow cytometry, or an injector-equipped luminometer.

### Western Blot Assay

Cells were harvested and disrupted in a radioimmunoprecipitation assay (RIPA) lysis buffer. Following normalization for protein concentration, the samples were separated using 12% SDS-PAGE electrophoresis, electro-transferred to nitrocellulose membranes (Bio-Rad), and probed with relevant primary antibody at 4°C overnight. Then, the membranes were incubated with secondary antibodies and detected with an enhanced chemiluminescence substrate (MEMD Millipore, Billerica, MA, USA). β-actin served as the loading control. The western blot assays for the detection of TCS-FLAG and β-actin used the following antibodies: monoclonal rabbit anti-FLAG primary antibody at 1:2,000 (Cell Signaling Technology, catalog #14793), monoclonal mouse anti-β-actin primary antibody at 1:2,000 (Beyotime Biotechnology, catalog #AF0003), and horseradish peroxidase-conjugated-labeled goat anti-mouse IgG secondary antibody at 1:2,000 (Beyotime Biotechnology, catalog #A0216).

### The rAAV2 Vector Transduction Assays *In Vivo*

Male 6- to 8-week-old wild-type C57BL/6 (B6) mice were purchased from the Shanghai Laboratory Animal Center (SLAC), Chinese Academy of Sciences, Shanghai, China. All mice were housed in an SPF animal facility at Fudan University, with controlled temperature and humidity and an artificial 12-hr/12-hr on/off light cycle. A total of 4–5 mice were housed per cage.

The rAAV vectors were intravenously injected via the tail vein into C57BL/6 mice. For the fluorescence reporter gene, mice livers were harvested at 8 weeks after vector administration, and thin sections from each hepatic lobe were mounted on slides and visualized under a fluorescence microscope. For the luciferase reporter gene, mice were weighed to calculate the volume of substrate, D-luciferin-K+ salt (Caliper Life Sciences, Hopkinton, MA, USA), according to a dose of 4 mg/(kilograms ⋅ day) of body weight, and anesthetized. The calculated volume of 5 mg/mL stock substrate solution was mixed with 100 μL PBS and intraperitoneally injected. *In vivo* bioluminescence images were acquired immediately over a period of 4 min using a Xenogen machine equipped with a cooled charge-coupled device camera (Xenogen, Alameda, CA, USA). Signal intensity was quantified using the camera control program, Living Image software, and was presented as photons per second per square centimeter per steridian (p/s/cm^2^/sr).

### Statistical Analysis

The results are presented as mean ± SD. The differences between groups were identified using a grouped-unpaired two-tailed distribution of Student’s t test. A p value < 0.05 was considered statistically significant.^32^

## Author Contributions

P.G., C.Y., and R.Z., performed the experiments. P.G., Y.F., X.M., and Q.W., designed the experiments and interpreted the data. P.G. and C.Y. wrote the manuscript.

## Conflicts of Interest

The authors declare that there is no conflict of interests.
